# Croup Treated by Passing Catheter into the Trachea by the Mouth

**Published:** 1881-10

**Authors:** J. Wilson Paton


### Croup Treated by Passing Catheter into the Trachea by the Mouth.

By J. Wilson Paton, M. D.

H. J., aged three years and ten months, had measles, the rash appearing Feb. 15, 1881. On the disappearance of the rash, a hard cough supervened, which gradually increased in severity till March 1st. I found him suffering from intense dyspnœa, quite unable to speak, and lips of a dark livid color. Cough was constant, brassy, and without expectoration. Respiration thirty-five per minute, cartilages of the ribs and sternum being drawn in at every effort to breathe, and crepitation existing over both lungs. Fauces healthy. Pulse 144, very weak. Having a No. 11 prostatic catheter, I determined to pass it into the trachea instead of performing tracheotomy. Watching an opportunity, while the tongue was depressed with a spoon, the catheter, curved a little more than usual, was passed into the trachea, during an attempted inspiration, and without the slightest difficulty. A severe struggle followed, lasting perhaps a minute or two, the face becoming purple, and the eyes staring with fully dilated pupils. The paroxysmal efforts to expel the tube being unsuccessful, a pretty full inspiration, partly through the tube and partly through the larynx, followed; about two ounces of frothy, bloody, and purulent mucus were ejected by the tube and the mouth; the livid color disappeared, and he lay down breathing easily through the tube. The presence of the tube did not prevent his swallowing milk, although sometimes a little of this was ejected from it during a cough. The tube was retained *in situ* by a strip of plaster; and the teeth were prevented from closing on it by means of a pear-shaped piece of hard wood. Six hours afterwards, he was much easier. Cough continued at intervals of ten minutes, and did not seem altered by the tube. Crepitation still existed over both lungs, an abundant muco-purulent secretion being passed both by tube and mouth. Hitherto he had been kept in a warm room; but now a bronchitis kettle maintained a moist temperature of 70°. Tube was removed without inconvenience after it had been in the trachea for eleven hours, as he had bitten it, and no air was passing through it. Shortly after symptoms of obstruction gradually reappeared. During the same evening, another ordinary gum elastic catheter No. 12 was introduced, only a slight momentary struggle and cough supervening. The presence of the tube led again to a very free expectoration of mucus. In the course of a few hours, the respirations and pulse became lower, and crepitation and dyspnœa ceased. When the tube had been in for forty-eight and a half hours, it was removed, and not again introduced. March 8, voice and chest sounds normal. This case would soon have ended fatally. Tracheotomy seemed inadmissible. The introduction of a tube into the trachea of a child is extremely easy and simple, and does not take more than two or three seconds.—*British Medical Journal.*

